# Human Cytomegalovirus Infections Are Associated With Elevated Biomarkers of Vascular Injury

**DOI:** 10.3389/fcimb.2020.00334

**Published:** 2020-07-09

**Authors:** Jennifer N. Styles, Reagan R. Converse, Shannon M. Griffin, Timothy J. Wade, Elizabeth Klein, Leena A. Nylander-French, Jill R. Stewart, Elizabeth Sams, Edward Hudgens, Andrey I. Egorov

**Affiliations:** ^1^United States Environmental Protection Agency, Office of Research and Development, Research Triangle Park, NC, United States; ^2^Department of Environmental Sciences and Engineering, Gillings School of Global Public Health, The University of North Carolina, Chapel Hill, NC, United States; ^3^United States Environmental Protection Agency, Office of Research and Development, Cincinnati, OH, United States; ^4^ORAU Student Services Contractor to US EPA, Chapel Hill, NC, United States

**Keywords:** cardiovascular disease, cytomegalovirus—HCMV, serum amyloid A, vascular injury, biomarkers, intercellular adhesion molecular-1 (ICAM-1), C-reactive protein, vascular cell adhesion molecule-1 (VCAM-1)

## Abstract

**Background:** Human cytomegalovirus (HCMV) infects ~50% of adults in the United States. HCMV infections may cause vascular inflammation leading to cardiovascular disease, but the existing evidence is inconsistent.

**Objective:** We investigated demographic predictors of HCMV infection and explored associations between HCMV infection status, the intensity of anti-HCMV Immunoglobulin G (IgG) antibody response, and biomarkers of inflammation and endothelial function which are known predictors of cardiovascular disease.

**Methods:** We conducted a cross-sectional study of 694 adults residing in the Raleigh-Durham-Chapel Hill, NC metropolitan area. Serum samples were tested for IgG antibody response to HCMV, and for biomarkers of vascular injury including soluble intercellular adhesion molecule 1 (sICAM-1), soluble vascular cell adhesion molecule 1 (sVCAM-1), C-reactive protein (CRP), and serum amyloid A (SAA). Associations between HCMV and biomarker levels were analyzed using two approaches with HCMV serostatus modeled as a binary variable and as an ordinal variable with five categories comprised of seronegative individuals and quartiles of anti-HCMV antibody responses in seropositive individuals.

**Results:** HCMV seroprevalence in the study population was 56%. Increased body mass index, increased age, female gender, racial/ethnic minority status, and current smoking were significantly associated with HCMV seropositivity in a multivariate regression analysis. HCMV seropositivity was also associated with 9% (95% confidence interval 4–15%) and 20% (0.3–44%) increases in median levels of sICAM-1 and CRP, respectively, after adjusting for covariates. The association between HCMV seropositivity and median levels of sVCAM-1 and SAA were positive but not statistically significant. Significant positive associations were observed between the intensity of anti-HCMV IgG responses and levels of sICAM-1 and sVCAM-1 (*p*-values 0.0008 and 0.04 for linear trend, respectively). To our knowledge, this is the first epidemiological study to show a relationship between anti-HCMV IgG responses and vascular injury biomarkers sICAM-1 and sVCAM-1 in the general population.

**Conclusion:** HCMV infections are associated with vascular injury and inflammation biomarkers in adult residents of North Carolina.

## Introduction

Human cytomegalovirus (HCMV) is a member of the herpes virus family. The virus can be transmitted through person-to-person contacts involving exchange of infected bodily fluids such as blood, saliva, breast milk, urine, oropharyngeal secretions, cervical, vaginal secretions, and seminal fluid; sexual contact is a frequent mode of transmission (Cannon et al., 2010). HCMV causes a long-term latent infection with periodic flare-ups. In a new HCMV infection, immunoglobulin M (IgM) is produced for ~3–6 months, after which HCMV-specific IgM can no longer be detected in serum (Prince and Lapé-Nixon, 2014). During this 3–6-month period, the immunoglobulin G (IgG) response is established and remains high throughout the life-long latency of an HCMV infection. Serum IgG tests are widely used to determine HCMV infection status. Anti-HCMV IgG response increases during reactivation or reinfection of HCMV (Mehta et al., 2000; Carlson et al., 2010; Prince and Lapé-Nixon, 2014). The IgG seroprevalence of HCMV in the US was 50.4 % between 1999 and 2004 according to the National Health and Nutrition Examination Survey (NHANES) (Bate et al., 2010).

Infection with HCMV is an important public health concern (Schleiss, 2008). It causes ~30,000 (0.7%) congenital infections annually in the US resulting in hearing loss, speech and developmental disabilities, or death for infected children (Ross and Boppana, 2004; Dollard et al., 2007; Martin et al., 2010). In adults with non-compromised immune systems, HCMV infection may occur without symptoms although some individuals may have transient mild mononucleosis-like symptoms (Lancini et al., 2014). Chronic HCMV infections have been associated with increased risks of cardiovascular disease (CVD), immune dysfunction, frailty, and depression (Simanek et al., 2014; Collins-Mcmillen et al., 2018). A proposed mechanism for the development of CVD as a result of HCMV infection is the increased conversion of prothrombin to thrombin, a clot forming agent, either directly or indirectly through increased inflammation (Popović et al., 2012). Previous *in vivo* and *in vitro* studies of CVD development due to HCMV infection have produced mixed results. High prevalence of HCMV viruses has been demonstrated in carotid atherosclerotic plaques (Yaiw et al., 2013). Some studies have also demonstrated positive associations between HCMV infection and various biomarkers of inflammation, endothelial function and vascular injury, such as C-reactive protein (CRP), soluble intercellular adhesion molecule 1 (sICAM-1), and soluble vascular cell adhesion molecule 1 (sVCAM-1). Several epidemiological studies have linked HCMV seropositivity with increased levels of CRP (Fateh-Moghadam et al., 2003; Betjes et al., 2007; Simanek et al., 2011); however, this association was not observed in two other studies, potentially due to the non-specific nature of inflammatory biomarker CRP (Simanek et al., 2014; Terrazzini et al., 2014). Three small (>100 participants) epidemiologic studies found associations between anti-HCMV IgG responses and increased levels of sICAM-1 and sVCAM-1 in serum samples in renal transplant patients and symptomatic individuals with primary HCMV infections, but not in asymptomatic healthy individuals (Nordoy et al., 2000; Eriksson et al., 2001; Lee et al., 2019). *In-vitro* experiments demonstrated that cellular adhesion molecules sVCAM-1 and sICAM-1 are released when endothelial cells are infected with HCMV (Popović et al., 2012). Meta-analysis of prospective studies demonstrated that HCMV infections were associated with an increased risk of CVD although some cohort studies included in this analysis detected no such association (Haider et al., 2002).

The objective of this study was to assess if HCMV infection affects four selected biomarkers of inflammation and endothelial function which are known predictors of cardiovascular morbidity and mortality: CRP, serum amyloid A (SAA), sVCAM-1, and sICAM-1. CRP and SAA are markers of acute inflammation. Chronically elevated CRP is linked with coronary artery disease however, elevated serum levels of CRP or SAA can also indicate other health conditions such as cancer (Zakynthinos and Pappa, 2009; Kaptoge et al., 2012; Baumann et al., 2017). VCAM-1 and ICAM-1 are biomarkers of endothelial function. These molecules are released into circulation by vascular endothelial cells in response to inflammation. Their useful functions include stable mediation of leukocyte adherence to the vascular endothelium and transmigration. However, elevated concentrations of VCAM-1 and ICAM-1 are linked with the development of atherosclerosis (Blankenberg et al., 2003; Galkina and Ley, 2007; Zakynthinos and Pappa, 2009).

## Materials and Methods

### Study Population

The ethical considerations of this study were reviewed and approved by the University of North Carolina Institutional Review Board (UNC IRB ref. # 12-2600). All participants provided written consent prior to data collection.

This study was comprised of two subsets of a cross sectional study of 703 adults (>18 years of age) in the Raleigh-Durham-Chapel Hill metropolitan area in North Carolina in 2013. The first sub-set was a convenience sample study (*n*_1_ = 352) recruited by the United States Environmental Protection Agency (US EPA) through local advertising (hereon referred to as US EPA subset). The second subset of individuals (*n*_2_ = 351) was recruited for the Sample Collection Registry for Quality Control of Biological and Environmental Specimens and Assay Development and Testing study protocol number 10-E-0063, ClinicalTrials.gov identifier NCT01087307 at the National Institute of Environmental Health Sciences (NIEHS), Research Triangle Park, NC (hereon referred to as NIEHS subset).

Serum samples and questionnaire data from the US EPA subset were collected as part of a cross sectional study of chronic infections conducted in 2013 at the US EPA Human Studies Facility in Chapel Hill, NC. For the NIEHS subset, recruitment and data collection were conducted at the NIEHS facility (Research Triangle Park, NC) in 2013. Aliquots of serum samples and questionnaire data from the NIEHS subset were shared with the US EPA. Collected serum samples were archived at −80°C until they were assayed for this study between late 2016 and early 2017.

The present study involved laboratory analysis of both subsets of samples for anti-HCMV IgG and analysis of biomarkers of vascular injury in the NIEHS subset. Samples from the US EPA subset have previously been analyzed using the same methods as the present study for multiple biomarkers of health including biomarkers of inflammation and endothelial function/vascular injury under research projects on the health effects of urban green spaces (Egorov et al., 2017, 2018).

### Questionnaire Data and Health Examinations

Participants reported their demographic information (age, gender, race, ethnicity) and smoking history. However, only the US EPA survey included information on education level. None of the surveys collected data on income or assets. There were differences in smoking history questions between NIEHS and US EPA surveys: “current smoker” was defined as a participant who had smoked in the past 24 h (NIEHS) or who reported being a current smoker (US EPA). For the purpose of the analysis in this study, smoking data from both surveys were combined into a single binary variable (smoker vs. non-smoker). Height and weight data were measured by clinical staff at the US EPA or NIEHS facilities. Body Mass Index (BMI) was calculated using the measured height and weight of participants and categorized using the World Health Organization (WHO) standard definitions for BMI categories: underweight, normal, overweight and obese (WHO, 2000).

### Laboratory Analysis

Serum samples were tested for IgG antibody response to HCMV using a commercially available ELISA kit (cat# ab108724, Abcam, Cambridge, MA) following manufacturer's instructions. Serum samples were diluted 1:100 in the manufacturer provided sample diluent prior to being assayed. Twenty percent of samples were assayed in replicate on the same or different plates. The acceptable level of the coefficient of variation was set below 20%; results not conforming with this requirement were excluded from statistical analysis. Blank corrected optical density (OD) values were divided by the plate specific cutoff value. Seropositive samples were defined as those with HCMV ratio to plate specific cutoff value >1.1 according to the manufacturer's instruction. Samples with inconclusive results (HCMV ratio to plate specific cutoff value 0.9–1.1) were retested. For a single sample that remained inconclusive after several retests the result was dichotomized using the average HCMV ratio of 1.0 as a cutoff.

Serum CRP, SAA, sVCAM-1, and sICAM-1 levels were analyzed using a commercially available electrochemiluminescence Meso Scale Discovery (MSD) quadruplex Vascular Injury Panel 2 microplate assay (cat# K15198D, MSD, Rockville, MD) following manufacturer's instructions. Serum samples were centrifuged at 2000 × *g* for 3 min and then diluted 1:1,000 in the assay diluent prior to analysis. Twenty percent of samples were assayed in replicate on the same or different plates. The acceptable level of the coefficient of variation was set below 20%; results not conforming with this requirement were excluded from statistical analysis. Responses were measured using an MSD QuickPlex SQ 120 instrument. Concentrations of the analytes were estimated from four-parameter logistic regression models fitted to serially diluted standards, as per manufacturer's instructions.

### Statistical Data Analysis

R (version 3.5.1) and SAS (version 9.4) statistical analysis software packages were used to analyze the survey and assay data. Shapiro-Wilks test for normality and quantile-quantile (Q-Q) plots were used to determine if logarithmic transformations were warranted. All four biomarkers of vascular injury (sICAM-1, sVCAM-1, CRP, and SAA), as well as BMI data were log-transformed to satisfy assumptions of normality. Race and ethnicity data were combined and dichotomized as non-Hispanic whites vs. all others. Race and ethnicity data were dichotomized this way because representation from other race categories in this study was not sufficient to make statistical comparisons. Missing values for covariates were imputed using the SAS procedure *mi* for arbitrary missing data based on the multivariate discriminant function method.

Next, a multivariate predictive logistic regression model was developed for HCMV seropositivity. Covariates that were significant predictors (*p* < 0.05) of HCMV seropositivity in this model (age, BMI, gender, race/ethnicity, smoking status) were also incorporated in subsequent regression models to analyze associations between HCMV serostatus and vascular injury biomarkers.

The association between HCMV serostatus and levels of vascular injury biomarkers were investigated using linear regression models adjusted for covariates (SAS procedure *genmod*). Two alternative approaches were used. First, a binary variable for HCMV serostatus was used to assess the effects of seropositivity (Model 1). Second, anti-HCMV IgG antibody data were categorized as seronegative (reference category); or as 1st, 2nd, 3rd, or 4th quartiles of antibody responses in seropositive individuals in order to investigate whether there was an association between the intensity of anti-HCMV IgG responses and the levels of vascular injury biomarkers (Model 2). All results were expressed as percent changes in the median biomarker values.

Additional analysis under the second approach was conducted using the SAS procedure *glm* with the *contrast* statement in order to test for a linear trend in the effect of the intensity of anti-HCMV IgG antibody response on vascular injury biomarkers. This analysis was conducted using the ordinal variable for anti-HCMV IgG response as described above and assuming equal distances between all adjacent categories.

## Results

Serum samples from 694 of 703 study participants (99%) were tested for anti-HCMV IgG response and all four biomarkers of vascular injury. Nine samples could not be analyzed due to low sample volumes. Seroprevalence of HCMV was 56% (Table 1). All samples retested on different plate lots produced consistent results. The age of participants ranged from 18 to 85 years. Almost two-thirds (62%) of participants were women. A majority of participants (51%) were non-Hispanic whites while 16% of participants declined to report their race. Almost a quarter (23%) of participants reported a habit of smoking. Almost two-thirds of participants (66%) were overweight or obese (BMI ≥ 25.0 kg/m^2^). Age, BMI, gender, smoking, and race/ethnicity status were significantly associated with HCMV seropositivity in exploratory univariate analysis (Table 1).

**Table 1:** Descriptive statistics for the study population.

**Factor**	**Level**	***N***	**%**	**HCMV seropositive**	**% seropositive**	**Chi-Square test *p-*value**
	All	694	100%	391	56%	N/A
**Age category**						
(years)	18–29	206	30%	91	44%	
	30–39	138	20%	82	59%	
	40–49	130	19%	87	67%	
	50–59	152	22%	92	61%	
	60–85	68	10%	39	57%	0.0005
**BMI**						
(kg/m^2^)	Underweight (<18.5)	7	1%	3	43%	
	Normal Weight (18.5–24.9)	226	33%	107	47%	
	Overweight (25.0–29.9)	209	30%	116	56%	
	Obese (≥30)	252	36%	165	65%	<0.0001^*^
**Gender**						
	Male	264	38%	128	48%	
	Female	430	62%	263	61%	0.001
**Race**						
	White	376	54%	153	40.7%	
	Black or African American	187	27%	139	74.3%	
	American Indian or Alaska Native	2	0.3%	1	50.0%	
	Asian or Pacific Islander	9	1.3%	4	44.4%	
	Other	17	2.4%	13	76.5%	<0.0001^**^
	Not reported	103	15%	81	78.6%	
**Ethnicity**						
	Non-Hispanic	558	80%	287	51.4%	
	Hispanic	34	5%	25	73.5%	0.01
	Not reported	102	15%	79	77.5%	
**Race/ethnicity**						
	Non-Hispanic whites	353	51%	138	39%	
	Other	231	33%	168	73%	<0.0001
	Not reported	110	16%	85	77%	
**Smoking habits**						
	Non-smoker	531	77%	273	51%	
	Smoker	159	23%	115	72%	<0.0001
	Not reported	4	0.6%	3	75%	

*N/A, not applicable*.

* *Cochran-Armitage trend test*.

** *Non-Hispanic White vs. all other races/ethnicities*.

For regression analysis, missing race/ethnicity data (*n* = 110) and missing smoking data (*n* = 4) were imputed as described in the Methods section. Results of a multivariate predictive regression analysis for HCMV seropositivity demonstrated that increased age, female gender, racial/ethnic minority status, smoking, and greater BMI were significant predictors of HCMV seropositivity (Table 2). Specifically, a 10-year increase in age was associated with 1.25 (95% CI 1.11–1.41) adjusted odds ratio (aOR) of seropositivity. Women had 2.06 (1.47–2.89) aOR of being seropositive compared to men. Non-white and/or Hispanic individuals had 2.90 (2.03–4.13) aOR of being seropositive compared to non-Hispanic whites. Current smokers had 2.32 (1.54–3.51) aOR of seropositivity compared to non-smokers. For regression analysis, BMI data were log_2_-transformed; thus, the effect estimate is expressed as 1.72 (1.02–2.88) aOR of seropositivity per doubling of BMI values.

**Table 2:** Effects of demographic predictors on HCMV seropositivity—adjusted odds ratios (OR) with a 95% confidence interval from a multivariate logistic regression model.

**Predictor**	**Level**	**Adjusted OR (95 % CI)**
Age (per 10-year increase)	NA	1.25 (1.11, 1.41)
Gender	Males	Reference
	Females	2.06 (1.47, 2.89)
Race/ethnicity	non-Hispanic whites	Reference
	All others	2.90 (2.03, 4.13)
Smoking	Non-smoker	Reference
	Smoker	2.32 (1.54, 3.51)
BMI (per 100% increase)	NA	1.72 (1.02, 2.88)

HCMV seropositivity was associated with significantly increased levels of sICAM-1 and CRP after adjusting for covariates (Table 3). Median adjusted sICAM-1 and CRP levels in seropositive individuals were 9.2% (4.5–15%) and 20% (0.3–44%) higher than in seronegative controls, respectively.

**Table 3:** Multiplicative effect estimates (95% confidence intervals) of HCMV seropositivity or the intensity of anti-HCMV IgG antibody responses on vascular injury biomarkers. All models were adjusted for age, BMI, smoking status, gender, and race/ethnicity.

**Model**	CMV serostatus, median value (range of values)^**^	**sICAM-1**		**sVCAM-1**		**CRP**		**SAA**	
		**Effect estimate (% change)**	***p*-value for trend**	**Effect estimate (% change)**	***p*-value for trend**	**Effect estimate (% change)**	***p*-value for trend**	**Effect estimate (% change)**	***p*-value for trend**
1. Seropositivity	Negative 0.38 (0.13–1.07)	Reference		Reference		Reference		Reference	
	Positive all 3.86 (1.10–10.97)	9.2 (4.1, 15)		2.2 (−2.3, 6.9)		20 (0.3, 44)		11 (−7.4, 32)	
2. Intensity of anti-HCMV IgG response	Negative 0.38 (0.13–1.07)	Reference		Reference		Reference		Reference	
	Positive 1st quartile 2.14 (1.10–2.68)	6.9 (−0.3, 15)		−0.9 (−7.1, 5.8)		28 (−1.5, 67)		29 (−0.4, 66)	
	Posit. 2nd quartile 3.26 (2.69–3.86)	8.7 (1.5, 17)		1.9 (−4.5, 8.6)		35 (4.4, 76)		−1.8 (−24, 27)	
	Posit. 3rd quartile 4.40 (3.87–4.86)	6.6 (−0.6, 14)		1.3 (−5.1, 8.2)		−3.1 (−26; 26)		−2.6 (−25, 26)	
	Posit. 4th quartile 5.63 (4.90–10.97)	16 (8.0, 25)	0.0008	7.7 (0.6, 15)	0.04	23 (−6.5, 62)	0.7	23 (−5.8, 61)	0.7

** *Test values represent the ratio of optical densities of the sample and microplate-specific control*.

In addition, greater intensity of anti-HCMV IgG antibody responses was significantly associated with increased levels sICAM-1 and sVCAM-1 (Table 3). Individuals in the top quartile of anti-HCMV IgG antibody responses had 16% (8.0–25%) and 7.7% (0.6–15%) higher median levels of sICAM-1 and sVCAM-1, respectively, compared to seronegative controls. Tests for linear trend using ordinal data on anti-HCMV IgG responses produced significant results for both sICAM-1 and sVCAM-1. Dose-response associations between the intensity of anti-HCMV IgG antibody response and biomarker level were not observed for CRP nor SAA.

## Discussion

We observed that HCMV seropositivity increased with greater age and BMI, and was more common among non-white and/or Hispanic individuals, women and smokers. Associations between HCMV seropositivity and increased age, race/ethnicity, and female gender are consistent with previously published results (Bate et al., 2010; Cannon et al., 2010; Simanek et al., 2011). Smoking, which is associated with lower socioeconomic status (Hiscock et al., 2012), has also been controlled for in previous studies of HCMV and CVD (Simanek et al., 2011).

Regression models were adjusted for the above socio-demographic covariates, and two models (previously described) were used to explore associations between anti-HCMV IgG responses and levels of vascular injury and inflammation biomarkers. HCMV-seropositive individuals had significantly higher levels of sICAM-1 and CRP than seronegative controls. In addition, significant linear trends were observed between the intensity of anti-HCMV antibody responses and levels of sICAM-1 and sVCAM-1, but not levels of CRP. The observed association between HCMV serostatus and SAA was positive but not statistically significant, and there was no linear trend for this biomarker.

Results of this study corroborated previously observed associations between HCMV seropositivity and serum CRP levels (Fateh-Moghadam et al., 2003; Betjes et al., 2007; Simanek et al., 2011). To our knowledge, this was the first epidemiological study in the general population to show associations between anti-HCMV IgG responses and vascular injury biomarkers sICAM-1 and sVCAM-1. Previous research has only demonstrated the effects of HCMV on these cellular adhesion molecules *in vitro*, as well as in renal transplant recipients and individuals with symptomatic primary HCMV infection (Nordoy et al., 2000; Eriksson et al., 2001; Popović et al., 2012).

VCAM-1 and ICAM-1 play important roles in the vascular endothelium (Zakynthinos and Pappa, 2009). HCMV has been detected in endothelial cells (Crough and Khanna, 2009; Terrazzini et al., 2014). It has been proposed that the disruption of endothelial cells by HCMV triggers induction of proinflammatory adhesion molecules sICAM-1 and sVCAM-1, which potentially contributes to atherosclerosis and atherothrombosis (Popović et al., 2012). Our finding that elevated HCMV IgG response is associated with increased serum levels of sICAM-1 and sVCAM-1 provides further support for this hypothesis.

Previous epidemiological research demonstrated that exposure to common air pollutants was associated with increased levels of CRP, sICAM-1 and sVCAM-1 (Bind et al., 2012). Air pollution has been shown to cause adverse impacts on the cardiovascular system including endothelial dysfunction, atherosclerosis and thrombosis leading to increased risks of cardiovascular morbidity and mortality (An et al., 2018; Tibuakuu et al., 2018). Therefore, the associations between HCMV and vascular injury biomarkers observed in this study suggest that HCMV infection may increase susceptibility to detrimental health effects of air pollution. Our results suggest that in future investigations of the relationship between air pollution and CVD risk, it would be beneficial to control for HCMV serostatus due to the high prevalence of HCMV infection.

This cross-sectional study involved collecting information on common socio-demographic confounders (variables that are associated with both HCMV and vascular injury biomarkers) which have been controlled for in previous studies of HCMV infections. However, unaccounted confounders might have biased the observed associations between HCMV seropositivity and increased levels of vascular injury biomarkers. Although our regression analysis adjusted for age (a common predictor of chronic herpesvirus infections) and other demographic factors, we cannot completely rule out that the observed associations were partially confounded by other herpesviruses, such as Epstein-Barr virus (EBV).

Anti-HCMV IgM serum antibodies were not measured in the present study due to the low likelihood of detecting active primary HCMV infections in this cross-sectional sample of adult US residents. Previous research demonstrated that the incidence rate in the US population ages 12–49 was ~1.6 infections per 100 susceptible person-years in 1988–1994 (Colugnati et al., 2007). Therefore, we are unable to conclude that all anti-HCMV IgG positive results were due to latent infection, even though it was the most likely stage of infection that the HCMV seropositive participants were experiencing.

The IgG test kits used in this study had high reported sensitivity (98%) and specificity (97.5%). Thus, false-positive responses and false-negative responses were unlikely to have a substantial impact on the observed associations of anti-HCMV IgG responses with vascular injury biomarkers.

The strength of our study lies in demonstrating positive associations between the intensity of anti-HCMV IgG antibody responses and vascular injury biomarkers sICAM-1 and sVCAM-1. Arguably, socioeconomic factors, such as income and education, are less likely to confound these associations. However, it has been shown that the HCMV reactivation rate increases with age and is higher in women than in men (van Boven et al., 2017). While age and gender were controlled for in regression models in this study, there might be hypothetical unmeasured behavioral or life-style confounding factors that affected both the frequency of HCMV reactivation and the levels of vascular injury biomarkers. Further research which follows participants over time is warranted in order to confirm an association between HCMV reactivation and levels of vascular injury biomarkers, and to elucidate the triggers of HCMV-related inflammation.

## Conclusion

We observed associations between HCMV serostatus, intensity of anti-HCMV IgG responses, and vascular injury biomarkers sVCAM-1, sICAM-1, and CRP, which are known predictors of adverse cardiovascular outcomes. These findings suggest that a HCMV infection may contribute to the development of atherosclerosis and CVD.

## Data Availability Statement

The datasets for this article are not publicly available because they contain personally identifiable information (PII) collected with institutional review board review and approval which included an informed consent process which indicated individuals would not be identified as a result of their participation. Requests to access these datasets should be directed to Timothy J. Wade, wade.tim@epa.gov.

## Ethics Statement

This study involving human participants were reviewed and approved by the University of North Carolina at Chapel Hill Institutional Review Board. The patients/participants provided their written informed consent to participate in this study.

## Author Contributions

JNS, AE, RC, SG, and TW designed the study. RC, EH, and ES performed sample processing and contributed to data collection. AE, RC, TW, EH, and ES organized the study. JNS, SG, and EK carried out the laboratory experiments. JNS and AE performed data analysis. JNS, AE, LN-F, JRS, and TW drafted the manuscript. All authors contributed edits and comments.

## Conflict of Interest

The authors declare that the research was conducted in the absence of any commercial or financial relationships that could be construed as a potential conflict of interest.
